# Putative role of non‐invasive vagus nerve stimulation in cancer pathology and immunotherapy: Can this be a hidden treasure, especially for the elderly?

**DOI:** 10.1002/cam4.6466

**Published:** 2023-08-17

**Authors:** Auwal Abdullahi, Thomson W. L. Wong, Shamay S. M. Ng

**Affiliations:** ^1^ Department of Rehabilitation Sciences The Hong Kong Polytechnic University Kowloon Hong Kong Special Administrative Region China

**Keywords:** cancer, immunotherapy, inflammation, regenerative rehabilitation, vagus nerve stimulation

## Abstract

Cancer is globally a disease of significant public health concern owing to its prevalence, and association with morbidity and mortality. Thus, cost‐effective treatments for cancer are important to help reduce its significant morbidity and mortality. However, the current therapeutic options for cancer such as chemotherapy, radiotherapy, and surgery may produce serious adverse events such as nausea, vomiting, fatigue, and peripheral neuropathy, especially in the long term. In addition, these therapeutic options may not be well tolerated by the elderly especially those who are frail. The current article is aimed at discussing an alternative therapeutic option, non‐invasive vagus nerve stimulation (VNS), and the roles it plays in cancer pathology and immunotherapy. The VNS does this by reducing oxidative stress via silent information regulator 1 (SIRT1); inhibiting inflammation via both hypothalamic–pituitary–axis (HPA) and the release of corticosteroid from the adrenal gland, and cholinergic anti‐inflammatory pathway (CAP), and increasing vagal activity which helps in the regulation of cell proliferation, differentiation, apoptosis, and metabolism, and increase chance of survival. Furthermore, it helps with reducing complications due to cancer or its treatments such as postoperative ileus and severity of peripheral neuropathy induced by chemotherapy, and improves cancer‐related fatigue, lymphopenia, and quality of life. These suggest that the importance of non‐invasive VNS in cancer pathology and immunotherapy cannot be overemphasized. Therefore, considering the safety of non‐invasive VNS and its cost‐effectiveness, it is a therapeutic option worth trying for these patients, especially in combination with other therapies.

## 1 INTRODUCTION

Cancer is a disease of the genome that arises from alterations in DNA signaling and metabolism, leading to uncontrolled division and survival of transformed cells.^1^, ^2^, ^3^ The alteration can be caused by many factors. One of the factors is the error in protein synthesis and DNA duplication, which results in metabolic waste accumulation over a long period of time.^4^, ^5^ Thus, given that there is an inherent error rate in DNA replication with increasing age, all multicellular organisms are at the risk of developing neoplasm that may result in cancer development with time.^6^, ^7^, ^8^

In addition, pathogenic organisms such as viruses, toxic chemical compounds, and radiation can also lead to cancer pathogenesis by promoting inflammation in the cells.^5^, ^9^, ^10^, ^11^, ^12^, ^13^, ^14^, ^15^, ^16^ Furthermore, reversible alterations in gene expression called ‘epigenetic changes’ can also cause neoplasm, which may lead to the development of cancer.^17^, ^18^

According to global cancer statistics, as of the year 2020, there were about 19 million new cases of cancers globally; out of which, nine million cases resulted in death.^19^ In addition, although, there are many types of cancers, there are 10 types of cancers that are recognized as major types of cancers.^20^ These include breast invasive carcinoma, glioblastoma multiforme, head and neck squamous cell carcinoma, bladder urothelial carcinoma, rectum adenocarcinoma, kidney renal clear cell carcinoma, lung squamous cell carcinoma, uterine corpus endometroid carcinoma, colon adenocarcinoma, and ovarian serous cystadeno carcinoma.^20^ However, despite the number and different types of cancers, they share several pathological processes.^21^, ^22^

The pathological processes they share are the capacity to convey information for cell growth and increase in its number for a long time; avoid genes that forcibly put an end to cell development and growth; resist cell death, enable unlimited replication of cells; and form new blood vessels and invade and migrate to other cells and tissues of the body.^23^ The capacity to convey information for cell growth and increase its number for a long time is caused by oxidative stress, avoidance of genes that forcibly put an end to cell development and growth, enabling unlimited replication of cells and formation of new blood vessels are contributed by inflammation, and invasion and migration to other cells and tissues is caused by inflammation and increased, and uncontrolled activity of the sympathetic nervous system.^22^, ^24^, ^25^, ^26^, ^27^, ^28^

Thus, some of the therapeutic targets for cancer are oxidative stress, inflammation, and sympathetic nerve activity. For these, chemotherapy, radiation therapy, and a host of other therapies are used.^29^, ^30^, ^31^ However, these therapies may produce serious side effects such as nausea, vomiting, fatigue, and peripheral neuropathy especially when they are used for a very long time.^32^, ^33^, ^34^ In particular, for the treatment of cancer in the elderly, the kind of therapies that should be given deserve special consideration.^35^ This is because, the risk of developing cancer and death thereof, increases with age.^36^ The reason for this, is the accumulation of mutation over the years.^37^ Notably, as humans age, unrepaired DNA causes genomic instability, which as earlier noted, results in cancer.^1^, ^2^, ^3^, ^38^ In addition, the types of treatment given may be associated with more adverse events. For instance, elderly people, especially those who are frail have an increased risk of mortality, morbidity, and complication due to surgery,^39^ and surgery is one of the interventions used for people living with cancers.^40^

In addition, since many people in the world are suffering from one or more cancers, and that the disease incidence increases with age, and it has high potential for morbidity and mortality, finding any therapeutic solutions especially those that are cost‐effective, will be welcomed with an open arm. One such therapeutic option that is nowadays being considered, is the vagus nerve stimulation (VNS).

## 2 VAGUS NERVE STIMULATION

Vagus nerve stimulation (VNS) is any method such as the use of electrical current, manual touch, mindfulness meditation, or deep breathing exercise to stimulate the branches of vagus nerve to help modulate its neurophysiological and visceral functions such as increasing the levels of norepinephrine (NE) and gamma amino‐butyric acid (GABA), and control of digestion, heart rate, and respiration.^41^, ^42^, ^43^, ^44^ The vagus nerve is the tenth cranial nerve, a major component of the parasympathetic nervous system, and the longest cranial nerve in the body.^45^, ^46^, ^47^ It performs both motor and sensory functions, as it is comprised of 80% afferent (sensory) fibers that carry information from the body to the brain, and 20% efferent fibers that carry information from the brain to the body.^41^, ^48^, ^49^ Thus, the nerve serves a two‐way means of carrying information from the body to the brain and vice‐versa, by meandering from the brainstem to the proximal two‐thirds of colon, giving off many branches to various organs and parts of the body such as the tongue, pharynx, heart, and gastrointestinal system in order to help maintain homeostasis.^49^ This wide distribution of the nerve throughout the body, makes it to have many important clinical correlations^46^; and as such, VNS is carried out to subserve the aforementioned roles and more.

Stimulation of the vagus nerve can be carried out directly via surgically implanted electrodes connected to it (invasive VNS); or indirectly via electrodes applied over the distribution of its peripheral branches in the skin, manual touch of its branch in the neck, and respiratory stimulation of its diaphragmatic branch (non‐invasive VNS).^42^, ^50^ However, the invasive method is more associated with adverse events such as cardiac arrhythmias, vocal cord palsy, dysphagia, taste disturbance after the surgery (metallic taste), atrial fibrillation, reduced oxygen saturation, and chest pain that may be serious compared to the non‐invasive VNS.^50^, ^51^, ^52^, ^53^ Thus, considering non‐invasive VNS in the treatment of cancer, may be a long‐awaited hidden treasure. This is because, nowadays, in cancer treatments, the use of less or non‐invasive treatment options such as the use of ablation therapy is gaining prominence.^54^, ^55^, ^56^

In addition, as noted earlier, some of the methods of administering non‐invasive VNS include electrical, manual touch, respiratory stimulation of the peripheral branches of the vagus nerve, and mindfulness meditation.^41^, ^42^, ^57^ For electrical stimulation, it relies on the cutaneous distribution of vagal afferents, either at the external ear (auricular branch of the vagus nerve) or at the neck (cervical branch of the vagus nerve).^49^, ^58^, ^59^, ^60^ However, the concha and inner tragus of the external ear are regarded as the most suitable stimulation areas for non‐invasive VNS.^49^ For the VNS using manual touch, the peripheral branch of the vagus nerve in the neck around the position of the carotid sheath is pressed or electrically stimulated.^41^, ^61^, ^62^ This is because, after the vagus nerve exited the brainstem, it coursed through the neck, around the carotid sheath area.^63^ For the respiratory stimulation of the vagus nerve, slow‐deep breathing is used to stimulate the diaphragmatic branch of the vagus nerve.^64^ For the mindfulness meditation, it is a stress‐coping technique that promotes relaxation and elevates heart rate variability (HRV), an indicator of stimulation of the activity of the vagus nerve.^57^

So far, several reports have shown the effects of non‐invasive VNS at improving functions such as motor function following stroke, control of seizures in epilepsy, cognitive function, and acute respiratory distress syndrome in Covid‐19.^52^, ^53^, ^65^, ^66^, ^67^, ^68^, ^69^ The aim of this article is to present the putative role of non‐invasive VNS in cancer pathology and immunotherapy.

## 3 PUTATIVE ROLE OF NON‐INVASIVE VAGUS NERVE STIMULATION IN CANCER PATHOLOGY AND IMMUNOTHERAPY

**FIGURE 1:**
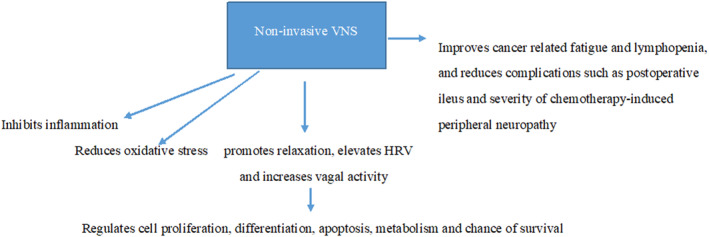
Mechanisms of treatment of cancer using VNS. Keywords: HRV, heart rate variability; VNS, vagus nerve stimulation.

Roles of non‐invasive VNS in cancer pathology and immunotherapy are emerging. In particular, several important mechanisms have been identified: oxidative stress reduction; inhibition of inflammatory response by targeting two inflammation pathways (the hypothalamic–pituitary–adrenal axis (HPA) and the cholinergic anti‐inflammatory pathway) and inhibition of sympathetic activity.^48^, ^70^, ^71^ See Table 1 for the summary of the potential VNS parameters for patients with cancer^72^, ^73^, ^74^, ^75^; and Figure 1 for the schematic representation of its mechanisms.

**TABLE 1:** Potential parameters for the treatment of cancer using vagus nerve stimulation (VNS).

Study	Study design	Population	Site of stimulation	Intensity
Reijmen et al.^72^	Experimental study (mice model of Lewis Lung carcinoma) and a randomized controlled trial (RCT) (patients with non‐small cell lung cancer)	Mice (mice model of Lewis Lung carcinoma) and Humans	For the experimental study, the electrodes were placed in the neck of the mouse lateral to the trachea and over the left cervical vagus nerve. For the RCT, the stimulation needle was placed in the ear tragus	For the experimental study, electrical stimulation (1 ms duration, 5 kHz, 12 V sine waves repeated at 25 Hz; impedance: 350 ohm) was delivered twice per day in the form of 3 successive 2‐min trains. For the RCT, The stimulation intensity was set at a pulse width of 200 μs and pulse frequency of 25 Hz
Dubeykovskaya et al.^74^	Experimental study	Mice	Two bipolar platinum electrodes were applied to the vagus nerve in the left subdiaphragmatic vagus nerve trunk	Electrical stimulation was delivered at 1 V, 2 ms, 5 Hz for 240 min by a stimulation module (STM100A)
Gierthmuehlen et al.^75^ [2023]	RCT protocol	Humans (patients with gastrointestinal cancers)	Left‐sided ear tragus	An above‐threshold stimulation with 25 Hz, 250 μs pulse width, and 28‐s/32‐s on/off paradigm will be delivered for 4 h throughout the day for 4 weeks
Salama et al.^73^	Clinical trial	Humans (patients with non‐small cell lung cancer)	The stimulation needle was placed in the triangular fossa of the external acoustic meatus, to stimulate the auricular branch of the vagus nerve	Stimulation was delivered at a stimulatory pulse duration of 200 μs ± 20% with an effective voltage /current of 2.3 mV/230 nA at 10 kX. The stimulation phase continued for 40 min followed by a 20‐min pause. The stimulation started 24 h prior to the operation and continued till the 4th postoperative day

### 3.1 Oxidative stress reduction

Oxidative stress is a pathological process that results from an imbalance between the production and accumulation of reactive oxygen species such as superoxide radicals, hydrogen peroxide, hydroxyl radicals, and singlet oxygen in the cells and the ability of the body system to detoxify them.^76^, ^77^ Vagus nerve stimulation (VNS) reduces oxidative stress via silent information regulator 1 (SIRT1), which is a histone of deacetylase of nicotinamide adenine dinucleotide (NAD^+^) that helps in the regulation of cell proliferation, differentiation, apoptosis, and metabolism.^71^, ^78^

### 3.2 Anti‐inflammatory effect

Inflammation characterizes cancer pathology.^16^, ^79^ It is defined as the cellular, tissue, organ, or system's response or defense against a foreign body such as pathogens, chemical compounds, and radiation.^80^ It results in cancer when it fails to resolve over a long period of time.^1^, ^81^ This is because, when inflammation persists for a very long period of time, prolonged tissue damage occurs, which in turn induces cellular proliferation, a precursor for cancers.^1^, ^2^, ^3^, ^82^

The pathological process through which inflammation results in cancer generally starts from the recognition of the foreign body by the cell surface pattern receptors, which is then followed successively by the activation of inflammatory pathways, release of inflammatory markers, and recruitment of inflammatory cells.^83^ The inflammatory pathways that are activated include mitogen‐activated protein kinase (MAPK), nuclear factor kappa‐B (NF‐κB), and Janus kinase (JAK)‐signal transducer and activator of transcription (STAT) pathways.^84^, ^85^, ^86^ Activation of these pathways in turn results in the activation of pro‐inflammatory cells such as the macrophages, and the release of inflammatory biomarkers such as the interleukin‐1 beta (IL‐1β), interleukin‐6 (IL‐6), tumor necrosis alpha (TNF‐α), and other inflammatory proteins and enzymes that eventually cause abnormal growth of the cells, damage, and death.^83^ Thus, arresting these pathological processes can be an important immunotherapeutic target.

Vagus nerve stimulation (VNS) is known to reduce systemic inflammatory response.^87^ This is because, through the use of its afferent and efferent pathways, the vagus nerve brings about an anti‐inflammatory effect that inhibits inflammation.^41^, ^70^ For that to occur, the afferent pathways will activate or regulate the hypothalamic–pituitary–axis (HPA) and release of corticosteroid by the adrenal gland.^70^ Similarly, the efferent pathways will regulate the cholinergic anti‐inflammatory pathway (CAP).^41^

#### 3.2.1 Activation/ regulation of HPA

The HPA is a complex part of the autonomic nervous system of neuroendocrine pathways that responds to negative feedback loops, involving hypothalamus, anterior pituitary gland, and adrenal gland to help maintain physiological homeostasis.^88^ It is the primary innate defense against inflammation.^89^ This is made possible because, the afferent fibers of the vagus nerve are endowed with interleukin‐1β (IL‐1β) receptors in the paraganglia which transmit sensory information to nucleus tractus solitarius (NTS), where neurons located in the A2 noradrenergic group are activated and then project information to the parvo‐cellular zone of paraventricular nucleus of the hypothalamus (PVH) around corticotrophin‐releasing factor (CRF)‐containing neurons.^90^ These CRF neurons then activate the release of adreno‐corticotrophin hormone by the hypophysis that will finally stimulate the release of glucocorticoids by the adrenal glands to decrease peripheral inflammation.^90^

Consequently, the HPA is the physiological basis for the use of hormonal treatments for cancer malignancies and, it is considered cytostatic because it restricts tumor development by limiting the hormonal growth factors acting through the direction of HPA, hormone receptor blockage, and limiting adrenal steroid synthesis.^70^ Interestingly, VNS also acts on the same pathway to maintain homeostasis.^70^ For instance, it has been shown that VNS resulted in the downregulation of the insulin catabolic process, which may reduce circulating blood glucose that is pro‐inflammatory.^91^, ^92^, ^93^

#### 3.2.2 The cholinergic anti‐inflammatory pathway (CAP)

The cholinergic anti‐inflammatory pathway (CAP) is a neural mechanism of inhibiting inflammation via the parasympathetic nervous system activity that influences the level of circulating tumor necrosis α (TNF‐ α) and other inflammatory biomarkers such as the interleukins, and endotoxins.^94^ The VNS can activate the CAP by stimulating the activation of vagal afferent which inhibits inflammation by reducing or suppressing the production and release of pro‐inflammatory cytokines and biomarkers such as the tumor necrosis α (TNF‐ α) and interleukin‐6 (IL‐6).^41^, ^45^, ^95^, ^96^ In particular, VNS may delay tumorigenesis through its action on acetylcholine (ACh) and the acetylcholine receptor, α7nAChR, since they are widely expressed in many types of immune cells.^97^ In addition, the sympathetic nervous system and the vagus nerve act in synergy, through the splenic nerve, to inhibit the release of tumor necrosis factor‐alpha (TNFα) by macrophages of the peripheral tissues and the spleen.^48^

Furthermore, VNS helps to suppress the activation of other inflammatory pathways such as the activation of NF‐κB.^98^ Consequently, non‐invasive VNS has been reported to surge CAP to suppress 1, 2‐dimethyhydrazine (DMH) induced colon cancinogenesis.^99^ This role is similar to that of the drug, Roflumilast, a selective phosphodiesterase‐4 inhibitor (PDE4).^100^ Furthermore, non‐invasive VNS stimulates tumor‐infiltrating lymphocytes.^72^ The tumor‐infiltrating lymphocytes are types of immune cells that help attenuate acute inflammatory response and kill cancer cells.^72^, ^73^

### 3.3 Inhibition of sympathetic activity

High vagal activity, which is indicated by heart rate variability (which reflects combined activity of parasympathetic and sympathetic tone on heart rate) is related to good prognosis and increased chance of survival in patients with various forms of cancers such as colon, pancreatic, lung, and breast cancers.^101^, ^102^, ^103^, ^104^, ^105^, ^106^, ^107^ This is because high vagal activity inhibits sympathetic nervous system activity, which is associated with decreased plasma level of TNF‐ α.^108^ In contrast, the absence of vagal nerve activity promotes tumor growth and reduces cell survival due to increased levels of TNF‐ α, which in turn promotes invasion and migration of cancer cells, one of the hallmarks of cancer pathology.^23^, ^109^, ^110^

## 4 OTHER ROLES OF NON‐INVASIVE VNS IN CANCER

Other ways through which non‐invasive VNS play a role in patients with cancer are also many. For instance, postoperative ileus was reported to reduce following low‐intensity non‐invasive VNS (25 Hz, 50 mA) that was given for 20 min prior to anesthesia in patients who had laparoscopic radical resection of colorectal cancer.^111^, ^112^ Similarly, it was also reported to help reduce the severity of peripheral neuropathy induced by chemotherapy.^32^ In addition, it helps improve cancer‐related fatigue, lymphopenia, and quality of life.^113^ Thus, the importance of non‐invasive VNS cannot be overemphasized.

Furthermore, the positive association between depression and anxiety and incidence of cancer and all‐cause mortality^114^; and the increased risk of depression among patients with cancer are also factors that can be considered as mechanisms through which VNS plays a role in the treatment of cancer.^115^, ^116^, ^117^ This is because VNS is used in the management of depression, which by implication helps improve a symptom associated with cancer and reduce the risk of developing it.^118^, ^119^ Similarly, cardiovascular diseases and cancer share many common risk factors such as smoking, metabolic syndrome, age, environmental toxins, and air pollution.^120^ Moreover, heart rate variability (HRV), which is the fluctuation in the time intervals between adjacent heartbeats serves as a measure of vagal tone that is used to indicate the overall level of vagal activity.^121^, ^122^ Thus, since HRV is a measure of cardiovascular health and an indicator of vagal activity, improving cardiovascular health with the use of VNS may help with positive outcomes during treatment of cancer.^57^, ^123^, ^124^, ^125^

## 5 IMPLICATION FOR RESEARCH AND PRACTICE

Acute inflammatory response is an essential and protective response in injured tissues, and it can at times restore the tissues to their preinjury state.^126^ In addition, cancer cells as well as surrounding stromal and inflammatory cells may engage in well‐orchestrated reciprocal interactions to form an inflammatory tumor microenvironment that is highly plastic.^15^ Thus, the use of non‐invasive VNS for cancer treatment should take advantage of the early stage of the disease and the plastic nature of the tumor microenvironment.

Another way non‐invasive VNS can be used for cancer treatment is by combining it with other therapies such as radiotherapy. This is because, such a combination has helped stimulate tumor‐infiltrating lymphocytes, types of immune cells that kill cancer cells.^72^ In addition, very often, immunotherapy is utilized as a part of a combinatory therapy along with other treatments like radiation, chemotherapy, remission surgery and so on.^127^

Although, the role of VNS in cancer pathology and immunotherapy is still ambiguous and requires further investigation^128^; however, its role may just be similar to that of the corticotrophic releasing hormone (CRH), which has an anti‐inflammatory effect when released from the brain, but pro‐inflammatory effect when released by the nerve endings at the site of the inflammation.^129^ This analogy was made because the vagus nerve consists of 80% afferent (sensory) fibers that carry information from the body to the brain.^41^, ^48^, ^49^ That way, it can sense peripheral inflammation and transmit action potentials from the periphery to the brain stem.^23^, ^130^ Thus, a proper understanding of the vagus nerve is required for understanding its pathophysiology and its potential roles in the treatment of diseases such as cancer.^131^

## AUTHOR CONTRIBUTIONS

**Auwal Abdullahi:** Conceptualization (equal); data curation (equal); funding acquisition (supporting); resources (equal); software (equal); writing – original draft (lead); writing – review and editing (equal). **Thomson W. L. Wong:** Conceptualization (equal); data curation (equal); funding acquisition (supporting); resources (equal); software (equal); writing – original draft (equal); writing – review and editing (equal). **Shamay S. M. Ng:** Conceptualization (equal); data curation (equal); funding acquisition (lead); resources (equal); software (equal); writing – original draft (equal); writing – review and editing (equal).

## Data Availability

Data sharing is not applicable to this article as no new data were created or analyzed in this study.
